# MSC-Derived Extracellular Vesicles in Tumors and Therapy

**DOI:** 10.3390/cancers13205212

**Published:** 2021-10-18

**Authors:** Tianjiao Luo, Juliane von der Ohe, Ralf Hass

**Affiliations:** Biochemistry and Tumor Biology Laboratory, Department of Obstetrics and Gynecology, Hannover Medical School, 30625 Hannover, Germany; luo_tj@yeah.net (T.L.); Ohe.Juliane.von.der@mh-hannover.de (J.v.d.O.)

**Keywords:** MSCs, extracellular vesicles, exosomes, tumor growth, microRNAs

## Abstract

**Simple Summary:**

Therapeutic functions of mesenchymal stroma-/stem-like cells (MSCs) are mediated predominantly through paracrine effects by the release of various different components. Upon recruitment of MSCs to damaged tissue sites or tumors, several bioactive molecules and organelles that are secreted by MSCs among others are cytokines, chemokines, metabolites, and extracellular vesicles including exosomes. The MSC-mediated cargo of released exosomes contains specific proteins and nucleic acids with varying regulatory microRNAs according to the tissue origin and the MSC microenvironment. These MSC-released exosomes are taken up by different target cells in damaged tissues to promote a regulatory network of tissue repair, including immune modulation and induction of angiogenesis. Conversely, in tumors, MSC-derived exosomes can confer predominant signals to suppress neovascularization and to relay further tumor-inhibitory effects. However, MSCs that adapted to the tumor tissue by mutual interaction with cancer cells progressively alter to an aberrant phenotype with the release of exosomes carrying tumor-supportive material.

**Abstract:**

Exosomes derived from mesenchymal stroma-/stem-like cells (MSCs) as part of extracellular vesicles are considered cell-free biocompatible nanovesicles that promote repair activities of damaged tissues or organs by exhibiting low immunogenic and cytotoxic effects. Contributions to regenerative activities include wound healing, maintenance of stem cell niches, beneficial regenerative effects in various diseases, and reduction of senescence. However, the mode of action in MSC-derived exosomes strongly depends on the biological content like different regulatory microRNAs that are determined by the tissue origin of MSCs. In tumors, MSCs use indirect and direct pathways in a communication network to interact with cancer cells. This leads to mutual functional changes with the acquisition of an aberrant tumor-associated MSC phenotype accompanied by altered cargo in the exosomes. Consequently, MSC-derived exosomes either from normal tissue-originating MSCs or from aberrant tumor-associated MSCs can confer different actions on tumor development. These processes exhibiting tumor-inhibitory and tumor-supportive effects with a focus on exosome microRNA content will be discriminated and discussed within this review.

## 1. Introduction

A variety of different cell types, including cancer cells and mesenchymal stroma-/stem-like cells (MSCs) also termed multipotent mesenchymal stromal cells or medicinal signaling cells [1,2], produce and release extracellular vesicles (EVs) packed with various cellular content. EVs represent membranous organelles generated under different physiological and pathophysiological conditions. 

Accordingly, the biological content of EVs changes significantly in disease, inflammation, and cancer [3]. Different EV populations include exosomes, microvesicles, apoptotic/necroptotic bodies, phagosomes, and damage-associated molecular patterns (DAMPs), which can be discriminated and separated by size, content, and origin [4,5,6]. However, EV preparations may be co-enriched by lipid-based non-vesicular structures, such as chylomicrons or very-low-, low-, intermediate-, and high-density lipoproteins [7]. Moreover, enrichment of EVs includes the risk of contamination with similar-sized viruses and viral compounds. 

A predominant EV type with significant impact on adjacent cell populations is represented by exosomes that mutually affect cellular metabolism. Exosomes are formed from multivesicular bodies of the endolysosomal pathway origin after budding of the late endosomal membranes [8,9]. These double membranous exosomes contain a plethora of different proteins, DNA fragments, and RNAs. Exosome-containing RNAs include mRNAs, circRNAs, tRNAs, long non-coding RNAs, and regulatory microRNAs (miRs), whereby, e.g., bone marrow-derived (BM-)MSCs and adipose tissue-derived (AD-)MSCs contain distinct sets of tRNAs and miRs [10].

Exosomes are released in vitro into the supernatant conditioned medium and are shuttled in vivo within the body in almost all biological fluids, including blood, interstitial fluid, urine, saliva, and cerebrospinal fluid [8,11], to alter the functionality of recipient cells [12,13]. The uptake of exosomes and EVs in general by appropriate target cells involves a variety of different mechanisms [4,14,15,16]. These include among others clathrin-dependent and -independent pathways, macropinocytosis, phagocytosis, lipid raft-mediated internalization, EV fusion with the target cell plasma membrane, and receptor-mediated endocytosis [14]. These processes underlie different biochemical and cell biological processes. Micropinocytosis is mediated by actin filament-driven plasma membrane protrusions that form an invagination to non-specific endocytose extracellular fluid and small particles, such as EVs. Likewise, phagocytosis represents an actin cytoskeleton-mediated mechanism for EV incorporation. Prerequisites of EV incorporation by plasma membrane fusion include destabilization of the interacting lipid bilayers by local acidic pH and overcoming high activation energy barriers [15]. The predominant entry of exosomes into target cells may be performed via receptor-mediated endocytosis. This requires either a or a corresponding receptor on the exosome surface to engage its designated counterpart on the uptaking cellular plasma membrane [16]. Specific factors engaged in this process are glycolipids; various glycoproteins, such as connective lectins and/or adhesion molecules (e.g., integrins, immunoglobulins); mucins; and heparan sulfate proteoglycans. Accordingly, receptor-mediated endocytosis represents a specific process as compared to the other EV uptake mechanisms.

Recent work has suggested a three-step mechanism for the internalization of EV cargo: (i) targeting of the acceptor cell by EVs; (ii) entry point and internalization of EVs into the acceptor cells; and (iii) delivery of EV content to the acceptor cell [4]. Most publications report EV entry and cargo delivery into cells through internalization. However, exosome-mediated cellular responses can also be induced only by receptor membrane-binding without requirement for internalization. For example, soluble Fas ligand and TNF-related apoptosis-inducing ligand (TRAIL) from exosomes can perform temporary adhesion to recipient cell membranes to relay juxtacrine or soluble intracellular signaling.

Nevertheless, molecular players remain to be elucidated that determine by which pathway different EV populations gain entry into a cell. Thus, the targeting of appropriate recipient cells by exosomes may include specific as well as non-specific and stochastic pathways. This hypothesis is substantiated by interspecies activities of exosomes, e.g., during the transfer of exosome content from human cells into mice with the mediation of biological responses [17]. Conversely, exosomes may be addressed not only to certain recipient cells but also to subcellular compartments and organelles, such as the endoplasmic reticulum [18] or the nucleoplasm [19]. Together, the different routing of EVs for internalization/cellular uptake may be highly specific or stochastic depending on the EV size, EV compositions, EV heterogeneity, the cargo to be delivered, distinct microenvironmental conditions, and target cell specificities. 

Small-particle exosomes display a diameter of approximately 20 to 200 nm containing a double membrane with distinct sets of proteins. These typical exosome marker proteins are determined among others by surface glycoproteins of the tetraspanin transmembrane-4 family, such as CD9, CD63, and CD81 (=TAPA-1 (target of the antiproliferative antibody 1)=tetraspanin-28) [12,20]. A further exosome marker can include ESCRT (endosomal sorting complex required for transport) proteins and associated factors, such as Alix [21] (Apoptosis-linked gene 2-interacting protein X, also known as programmed cell death 6-interacting protein), that recruits ESCRT-III proteins to endosomes and contributes to the sorting of tetraspanins to exosomes. Moreover, the ESCRT-I tumor susceptibility gene 101 protein (TSG101) plays an important role in EV/exosome biosynthesis besides additional multifaceted biological functions, e.g., in endosomal trafficking [22]. 

**Figure 1:**
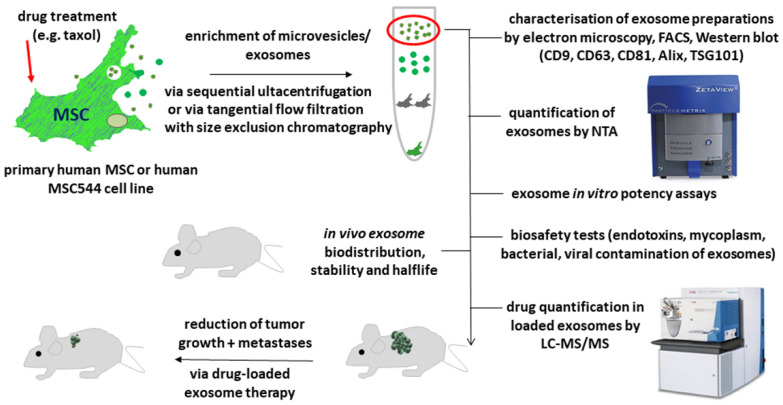
Drug-loading of human MSC-derived exosomes for in vivo antitumor application involves several steps of isolation and characterization. Primary human MSCs or the permanently growing human MSC544 cell line can be incubated with sub-lethal drug concentrations (e.g., 10µM taxol for up to 24 h) for incorporation of the drugs, packing into multivesicular bodies, and subsequently releasing drug-loaded exosomes into the conditioned medium. After removing the drugs by several washes and incubating the MSCs in drug-free and serum-free medium for a further 24 h, a microvesicular fraction including exosomes that are released into the conditioned medium can be enriched by different methods as suggested by protocols of Thery et al., together with the International Society of Extracellular Vesicles (ISEV) [29,30] and updated 2018 standards [31]. Enriched EVs/exosomes require appropriate characterization by various different methods. Analysis by transmission electron microscopy should demonstrate the typical exosome ultrastructure of 20 to 200 nm and rounded bodies with a double membrane. In addition, exosome marker proteins need to be detected by flow cytometry (FACS) or by Western blots. Moreover, nano-tracking analysis (NTA) confirms the average diameter of the exosome fraction and determines the exosome concentration and the electric field mobility (zeta potential) as one of the quality markers. Further exosome analysis by liquid chromatography coupled with double mass spectrometry (LC-MS/MS) quantifies the amount of drugs packed into the exosomes by the MSC. Several biosafety tests are required to exclude, e.g., viral contamination since viruses or viral particles are of a similar size and may therefore be co-enriched. The drug-loaded exosomes can be applied to potency assays, such as determination of successful cell killing activity with several different cancer cell populations in a chemosensitivity assay system. Based on the promising results of the chemosensitivity assays, appropriate exosome fractions can be used in tumor-bearing animal models to study effects on tumor growth and distal metastases. Alternatively, modified exosome fractions can be applied in vivo to determine different routes of delivery and to monitor a variety of important parameters, such as biocompatibility, biodistribution, dosage, pharmacokinetics, half-life, and stability.

Exosomes can be enriched and isolated by different methods, such as sequential ultracentrifugation, tangential flow filtration, and size exclusion chromatography (Figure 1). These nanovesicles are exchanged among cells in most areas of the body including normal, damaged, and tumor tissues. Amid the different cell types within the tumor microenvironment (TME) are the MSCs, strongly interacting with cancer cells. MSCs play an important role in the TME architecture and the TME interaction network. This cellular interplay may additionally include interactions with various different types of bacteria present in the TME. Indeed, previous studies have demonstrated that tumor-associated cells of distinct tumor entities including breast, lung, ovary, pancreas, melanoma, bone, and brain tumors carry a diverse microbiome displaying selective signatures [23]. The tumor-associated microbiome can modify tumor development and immune cell properties, particularly in certain gastrointestinal tumors [24]. In a colitis-associated colon cancer model, MSCs were involved in the regulation of gut microbiome imbalances accompanied by a reduced appearance of cancer cells [25]. While MSCs can confer antimicrobial activities in vivo [26,27] by involving pathways via indoleamine 2,3-dioxygenase [28], these capabilities could be altered within a neoplastic microenvironment. Thus, the tumor-associated microbiome contributes to the communication network of MSCs and derived exosomes with neighboring cells in the tumor tissue. Accordingly, the microbiome may complicate this orchestration of cellular interactions, being responsible for significant functional alterations, although detailed data remain to be elucidated.

With respect to MSC heterogeneity, the majority of MSCs within an interdependently mixed population displays stroma-like features while minor subtypes can exhibit stem-like properties of self-renewal, long-term maintenance of telomerase activity, and differentiation capacity at least along certain phenotypes of the mesodermal lineage [32]. Most of these mixed MSC subtypes share a common expression pattern of distinct surface markers, such as CD13, CD29, CD44, CD73, CD90, CD105, and CD166 with concomitant absence of at least CD14, CD31, CD34, and CD45 [32,33,34,35,36].

In the organism, MSCs preferentially reside in perivascular niches of nearly all kinds of human tissues [37,38] and can be activated to exhibit certain functions according to their tissue-specific origins. MSCs are predominantly recruited to sites of damage and injury to release exosomes with bioactive components and promote immune modulation, neo-vascularization, and tissue repair. Thereby, MSCs and their derived exosomes interact with various different cell types, such as immune cells, endothelial and mesothelial cells, pericytes, fibroblasts, and adipocytes [39]. 

During cellular interactions, MSCs release exosomes that contribute to various regenerative mechanisms and wound healing [40]. MSC-derived exosomes promote de novo skin tissue regeneration in response to cutaneous wound healing by enhancing re-epithelialization and dermal angiogenesis [41]. Repair activities of MSC-derived exosomes also include healing of fractured bones, whereby the data suggested that bone repair might be mediated in part by distinct miRs as exosome components [42]. Moreover, MSC-derived EVs/exosomes contribute to a reduction of senescence. Thus, BM-MSC-derived EVs from young mice extended the life span and improved health parameters in senescent animals, suggesting that MSC-derived EVs/exosomes slow the progression of aging and diseases driven by cellular senescence [43]. This heterochronic system to promote rejuvenation is also observed in exosomes released from initial passages of neonatal MSCs, such as umbilical cord-derived (UC-)MSCs. Interestingly, exosomes released from the permanently growing human MSC544 cell line [44] also reduced senescence and contributed to retrodifferentiation, providing a reproducible source of highly effective senotherapeutics.

MSCs and derived exosomes are also involved in stem cell homeostasis. In the bone marrow, for example, MSCs in orchestration with other somatic populations contribute to various stem cell niches and to the maintenance of tissue homeostasis [45] to enable self-renewal and expansion of hematopoietic stem cells and their immunological progeny [46,47]. 

Similar functionalities of MSCs may apply to pathophysiological conditions. Invasive tumor growth causes local tissue injuries and damaged lesions with the attraction of MSCs to this pro-inflammatory environment. Among the various cell populations within the TME, MSCs communicate with cancer cells by various direct and indirect pathways [48,49]. Direct cellular communications include (i) conexin-based gap junctional intercellular communication [50], (ii) notch receptor signaling involved in self-renewal and amplification of cancer stem cells (CSCs) [50,51], (iii) formation of F-actin-enriched nanotubes for the exchange molecules and/or small organelles [51,52], (iv) exchange of large cell membrane patches by trogocytosis [51,52], or (v) complete cell fusion between MSCs and cancer cells with the generation of new functional hybrid cancer cell populations [53,54,55]. Indirect interactions between MSCs and cancer cells are relayed by exchange of bioactive molecules or via EVs like exosomes. 

**Figure 2:**
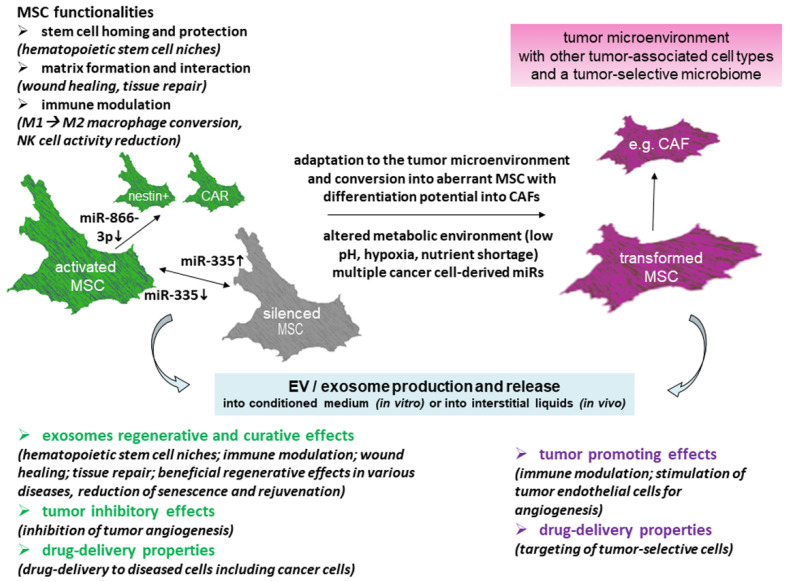
MSCs display several functionalities within the body. These are conferred predominantly after MSC-mediated release of exosomes following activation, e.g., by altered expression levels of miR-335 [68]. MSC-derived exosomes represent a heterogeneous population of vesicles that are equipped with different biological contents, including a large variety of miRs. Thereby, exosomes from normal MSCs can display regenerative, curative, and tumor-inhibiting effects. Following close interactions with cancer cells, however, MSCs can progressively develop along an aberrant tumor-associated phenotype. Accordingly, these tumor-associated MSCs release exosomes with altered cargo, eventually exhibiting tumor-supporting properties. Thus, MSCs can release exosomes with divergent functionalities depending on the microenvironment and the influence of local cellular interactions (scheme adapted from [48]).

While MSCs and cancer cells including cancer stem cells can secrete extracellular vesicles, mutual exchange of exosomes can alter both functionalities. Thus, tumor-derived exosomes can modify MSCs into supportive carcinoma-associated (CA-) MSCs or induce differentiation into cancer-associated fibroblasts (CAFs) [50,52,56,57] (Figure 2). In certain tumor entities, such as breast cancer [58] or colorectal carcinoma [59], CAFs have been associated with poor prognosis while the cells can remodel the extracellular matrix and the aberrant vasculature of tumors [60]. Vice versa, metabolic effects by exosomes from heterogeneous MSCs contain various unique factors displaying distinct functionalities in tumors [61,62,63]. During location in a tumorigenic microenvironment, cellular interactions of MSCs also contribute to immune modulation [64], tumor angiogenesis, and alteration of a large variety of cancer cell properties [48,51,57,65,66,67]. 

Together, MSCs and particularly their derived exosomes transport various bioactive molecules to the cellular environment and play an important role in regenerative medicine. On the other hand, MSC-derived exosomes take part during the development of various types of tumors [69,70]. It is therefore of interest to discriminate these different phenomena of MSC-derived exosomes and to assign certain exosome content, such as miRs, to distinct functionalities during tumor development.

## 2. Differential Effects of MSC-Derived Exosomes on Tumor Growth 

MSCs are recruited to tumor sites by microenvironmental changes, such as elevated acidification, nutrient deprivation, and increasing hypoxia. The accumulation of MSCs within the tumor stroma is also supported by increasing cytokine concentrations of fibroblast growth factor-2, monocyte chemotactic protein-1 (=CCL2), CCL5, and stromal cell-derived factor 1 (=CXCL12). Moreover, the pro-inflammatory cytokines tumor necrosis factor-α and interleukin 6 are produced by cancer cells and populations of the TME [71,72]. 

During recruitment, MSCs release exosomes that act as paracrine mediators. Thereby, exosomes transfer signal molecules, such as miRs, to other cell populations within the TME. These exosome-mediated miRs can contribute to the regulation of tumor growth by modulating cellular pathways including angiogenesis and immune control. Previous work has demonstrated the uptake and incorporation of MSC-derived exosomes by different breast and ovarian cancer cells. The associated acquisition of new cancer cell properties revealed an induction of matrix metalloproteinase-2 and ecto-5′-nucleotidase activity contributing to alteration of the tumor stroma [67]. Conversely, MSC-released exosomes can also mediate and activate signal pathways, eventually leading to tumor growth inhibition and prevention of metastases [73].

Consequently, MSC-derived EVs/exosomes can affect cancer cells and the TME by changing tumor growth in different and sometimes opposite directions:

(1) Promotion of tumor growth and metastasis;
(2) Moving cancer cells into quiescence/dormancy;
(3) Leaving tumors unaffected; and
(4) Inhibition of tumor growth.

These divergent and controversial findings about the functionality of MSC-derived exosomes may be attributed at least in part to the heterogeneity of the originating MSC populations themselves. The heterogeneity of these stroma-/stem-like cells is strongly influenced by the activation state of MSCs (Figure 2) and the external milieu. For example, the growth of MSCs on rigid/stiff in contrast to soft surfaces changes cytoskeletal functions and differentiation capacity [74] and thereby alters exosome release and content. Moreover, several physico-chemical factors within the microenvironment, such as low/high pH, hyperoxia/hypoxia/anoxia, and low/high ion gradients, modify MSC behavior and secretory activity for exosome production. Variation in these conditions can enable a growth advantage of distinct MSC subpopulations with either increasing heterogeneity or clonogenic convergence [75].

Significant stimulatory changes within the micromilieu can alter MSC properties and their derived exosomes. The activation status of MSCs partially depends on the type, threshold, and synergy of local stimuli. These effects can lead to controversial functionalities of either tumor promotion or tumor inhibition that can occur simultaneously at different sites within the same tumor tissues. These areas of opposite MSC functionalities may be compartmentalized, whereby the availability of specific stimuli remains limited to small regional parts within the MSC vicinity. Depending on the tumor compartment and the present localization, MSCs can be heterogeneously activated, e.g., to confer a reduced tumor growth in certain parts of the tumor tissue while in other tumor parts, MSCs receive different signals for transmitting stimulation of tumor growth [49,51]. Thus, the present balance of appropriate local stimuli determines tumor-inhibitory or tumor-promoting effects of MSCs. According to dynamic alterations of stimuli within parts of the TME, tumor-inhibitory MSCs and derived exosomes can be converted to a tumor-silencing (dormancy-inducing) or tumor-promoting MSC phenotype and vice versa. This suggestion underscores the role of MSC-derived exosomes in tumor plasticity [76] and is also in agreement with inhomogeneous tumor growth detectable in a variety of different tumor tissues. However, more studies on MSC-derived exosomes are required to elucidate molecular signaling pathways involved in the regulation of these controversial effects during tumor development.

Following inhibition or promotion of tumor growth MSC-derived exosomes can modulate the function of recipient cells [13,77]. This depends on the exosome content, such as the composition of several miRs that can vary under different conditions [78,79]. Accordingly, MSC-derived exosomes can transmit opposite signals for either promotion or inhibition of tumor growth in the same tumor type associated with distinct miR subsets (Table 1).

The data in Table 1 suggest that regulation of tumor growth in a supportive or inhibitory direction depends in part on different levels of certain miRs in MSC-derived exosomes. 

For discriminating different effects of MSC-derived exosomes on tumors, consideration should also be given to progressive changes of MSCs. Homing of MSCs to tumor sites has been suggested in response to chemotactic proteins emitted by cancer cells (see above). Following attraction to neoplastic tissues, normal tissue-associated MSCs progressively convert to a tumor-associated phenotype or they differentiate into CAFs (Figure 2) [90,91]. Tumor-associated MSCs regulate the survival, proliferation, migration, and drug resistance of cancer cells and participate in the formation of cancer stem cell niches (CSCNs) [49,92]. Moreover, tumor-associated MSCs in contrast to normal tissue-associated MSCs were found to secrete significantly more specific cytokines and metabologens, such as bone morphogenetic proteins [93]. These compounds stabilize tumor tissue at primary and metastatic sites, contribute to chemoresistance, and promote cancer stemness by the secretion of a specific set of paracrine factors [49,94].

The significant differences between normal tissue-associated MSCs and tumor-associated MSCs simultaneously affect the equipment and function of exosomes derived from these two MSC types. Indeed, in multiple myeloma exosomes from tumor-associated BM-MSCs, transferred low or undetectable levels of the tumor-suppressive miR-15a with the consequence of increased tumor burden together with elevated dissemination to distant bone marrow niches. By contrast, normal BM-MSCs carrying normal miR-15a levels in secreted exosomes contribute to inhibition of multiple myeloma tumor growth by reducing cancer cell dissemination (Table 1) [87]. Together, the data suggest differences in exosome content, such as miRs derived from originating normal or tumor-associated MSCs that can display opposite functionalities on tumor growth.

Similar effects may apply to the vascularization of normal and tumorigenic tissues. In normal tissues, MSC-mediated release of exosomes predominantly display pro-angiogenic miRs and associated properties. Thus, Gong et al. found a direct stimulation of human umbilical vein endothelial cells (HuVECs) by examination of tube-like structure formation and spheroid-based sprouting. Predominant pro-angiogenic miRs in MSC-derived exosomes transferred to HuVECS include miR-30b, miR-30c, miR-424, and let-7f [95]. Of interest, MSC-derived exosomes carrying let-7f, miR-145, miR-199a, and miR-221 also confer antiviral capacity, particularly for hepatitis C infections [96]. Moreover, pro-angiogenic effects of miR-132 containing MSC-derived exosomes were detected in myocardial ischemia [97]. 

Opposite effects, however, were observed in tumorigenic tissues. Human MSC-derived exosomes from marrow aspirates of healthy donors can suppress in vitro angiogenesis in breast cancer cells via miR100 by modulating the mTOR/HIF1α/VEGF signaling pathway [98]. Inhibition of angiogenesis in breast cancer cells was also documented in further studies, demonstrating a downmodulation of VEGF expression by murine BM-MSC-derived exosomes [99].

These dual effects of enhanced vascularization during repair of non-tumorigenic tissue damage in contrast to tumor suppression by inhibition of vascularization in tumor tissues are suggested to be properties of normal MSC-derived exosomes. 

## 3. MSC-Derived Exosomes Promote CSC Development in Associated Cancer Stem Cell Niches 

A CSCN physiology requires a variety of structural and cellular components to provide a local compartment for maturation and propagation of cancer stem progenitor and CSCs. For sufficient supply with nutrients and oxygen, certain CSCNs are localized in the vicinity of vascular structures. These specified structural elements, soluble factors, and released MVs/exosomes enable cell-to-cell interactions with adjacent cell types of predominantly stromal origin contributing to cancer stem cell maintenance [92].

Multiple CSCNs can be established and dismantled within the tumor tissues to regulate proliferation, apoptosis resistance, and maintenance of stemness. Various different models hypothesize CSC generation. These include concepts for tumor-initiating cells in a hierarchical [100,101] tumor model, by stochastic processes [102,103], or CSC development via a retrodifferentiation program [104]. In addition, new cancer cell populations carrying stem-like properties can also arise from cancer cell fusions with MSC or other cell types, whereby EV/exosome exchange can play an important role [52,105]. 

Thus, MSC-derived exosomes dynamically change interactions within a CSCN with the potential for tumor promotion and stabilization of a CSC pool [106]. In particular, MSC-derived exosomes activated the Hedgehog signaling pathway in recipient osteosarcoma and gastric cancer cell lines and induced tumor progression. Moreover, MSC-EVs originating from either murine or human bone marrow promote breast cancer cells to progressively retrodifferentiate and survive as CSCs in a dormancy-like state within the bone marrow [107]. Furthermore, BM-MSC-derived exosomes increase the population of CSCs in different colon cancer cells via transfer of miR-142-3p [108]. Collectively, MSC-released exosomes provide paracrine effects to support CSCN structures for the maintenance and progression of CSCs.

## 4. Exosomes from Tumor-Associated MSCs as a Tumor Biomarker

Cancer cell-derived exosomes can alter adjacent cells with a normal phenotype to adapt to the TME and contribute to tumor growth [109]. Moreover, certain exosomal adhesion molecules from cancer cells may direct metastatic development. Previous work has demonstrated that the integrins α6β4 and α6β1 in cancer cell-derived exosomes contributed to association with lung metastasis, while exosomal integrin αvβ5 was linked to liver metastasis [110]. Tumor-supportive functions from cancer cells can be acquired by MSCs. For example, exosomes from atypical teratoid/rhabdoid tumor-associated MSCs after conversion to an aberrant phenotype contained high levels of miR-155, which could cause inhibition of the tumor suppressor gene SMARCA4 as a direct target of miR-155 associated with enhanced migratory capability of atypical teratoid/rhabdoid tumors. This is in concert with the vulnerability of SWI/SNF chromatin remodeling genes in this tumor type and the related small cell hypercalcemic carcinoma of the ovary [111].

Aberrant regulation of metabolism in tumor-associated MSCs or tumor-differentiated CAFs display an altered pattern of bioactive compounds also released by exosomes. These metabolic changes in exosomes and alterations in the amount of components provide selective signatures of certain proteins and RNAs as a useful diagnostic tool for tumor biomarkers. Thus, deregulated miR patterns in exosomes from tumor-associated MSCs may represent biomarkers, e.g., for gastric cancer [112]. 

## 5. MSC-Derived Exosomes as a Vehicle for Drug Delivery in Clinical Application 

The use of MSC-derived exosomes as a cell-free system is well tolerated in patients (e.g., during the treatment of graft versus host disease) and may therefore provide an inert application system to transport and deliver antitumor cargo. For instance, taxol-loaded MSC-derived exosomes can operate as a vehicle to target breast cancer cells [17]. However, complex structures of the extracellular matrix within the tumor tissue together with interacting cells and the microbiome of the TME may also build a barrier for chemotherapeutic interventions to mimic certain drug resistance [113]. Several clinical trials are underway to date using MSC-derived exosomes for various antitumorigenic applications, e.g., for the treatment of advanced colorectal cancer, pancreatic cancer, and breast cancer [77].

MSC can be drug-stimulated or engineered to encapsulate appropriate compounds, e.g., antitumor cargo into exosomes. These disease-directed MSC-derived exosomes may qualify for appropriate therapeutic approaches. However, beside the disease-oriented modifications, the exosomes also confer their own biological activity based upon the diverse equipment with proteins, metabolites, and nucleic acid. For example, different miRs content can promote antagonizing effects in tissues and tumors (see above). Therefore, these dual modes of action (own biological effects of exosomes and applied modifications) need to be considered in clinical applications. The resulting functional discriminations should avoid unwanted or unfavorable effects on patient safety. Therefore, a variety of pre-clinical tests and settings still need to be addressed before pharmaceutical exosome manufacturing and routine clinical applications, whereby data from the ongoing clinical trials may add valuable information.

Protocols for the production and isolation of MSC-derived exosomes have been developed for good manufacturing practice grade [29,31] that necessitate adaptation to an industrial up-scaling. However, due to the heterogeneity of EVs/exosomes with respect to content and functionalities that are accompanied by diverse effects on the target cells, there is still a need for the establishment of reproducible standards. Beside basic characteristics (electron microscopy, tetraspanin expression, NTA, etc.) (see above), such standardization of exosomes features also applies to therapeutic biosafety and particularly to potency assays for comparable bioactivities, dosage, and pharmacokinetics as outlined in Figure 1.

Further aspects of consideration include the amounts of exosomes administered to patients for a distinct disease. This requires validation of the results on the biodistribution, stability and half-life of the disease-directed MSC-derived exosomes (Figure 1). Studies in a pancreatic cancer model address some of these questions and suggest possible mechanisms for maintenance of exosomes within the circulation and preventing uptake by monocytes/macrophages [114,115]. Further animal xenograft models can provide additional supportive data by addressing questions about a local or a systemic application form (e.g., oral or intravenous) and the frequency of repetitive exosome injections. 

## 6. Conclusions

MSC-derived EVs/exosomes provide a variety of promising opportunities for clinical applications by using novel MSC-based cell-free strategies. This applies to MSC primary cultures but particularly to the long-term growing human MSC544 cell line with constantly reproducible properties. In damaged tissues and various organ-related or degenerative diseases, MSC-derived exosomes can induce repair activities and promote regenerative potential. These EVs/exosomes can also serve as highly effective senotherapeutics by contributing to retrodifferentiation, rejuvenation, and longevity. However, there is still a need to standardize various parameters for reproducible clinical applications of exosomes.

As a diagnostic tool, exosomes can unravel specific patterns of biomolecules, such as the early appearance and detection of certain proteins or miR profiles during various diseases including tumor development. Due to the emerging tropism of MSCs towards tumors, the MSC-derived exosomes can be used in a tumor-therapeutic approach as a vehicle to selectively and specifically address and deliver antitumor components to cancer cells. Moreover, MSCs can be educated to release curative cargo such as specific proteins and miRs via exosomes to address damaged/dysfunctional cells and diseased tissues for enabling therapeutic requirements. However, the complexity of exosome interactions within different orchestrating populations and a dynamically changing metabolic microenvironment requires careful discrimination and evaluation.

## Figures and Tables

**Table 1:** MSC-derived exosomes contain miRs with tumor-promoting and tumor-inhibitory effects.

Tumor Type	Effect on Tumor Promotion	Effect on Tumor Inhibition	miR	References
glioma	proliferation and clonogenicity of tumor-initiating glioma stem-like cells		1587	[80]
		inhibition of tumor growth and down-modulation of AGAP-2	199a	[81]
prostate cancer	tumor progression via suppression of RHPN2		205	[82]
		tumor suppression via down-modulation of trefoil factor 3	143	[83]
breast cancer	induction of chemoresistance via S100A6 expression		21-p5	[84]
		tumor dormancy	222/223 23b	[85] [86]
multiple myeloma	enhanced tumor growth and dissemination		reduced 15a	[87]
		reduced tumor growth and dissemination	normal 15a	[87]
lung cancer non-small cell lung cancer	enhanced proliferation by inhibition of PTEN		410	[88]
		inhibition of tumor growth by targeting CCNE1 and CCNE2	144	[89]

## Abbreviations

BMT	bone marrow transplantation
CAFs	cancer-associated fibroblasts
CSCs	cancer stem cells
CSCN	cancer stem cell niche
ESCRT	endosomal sorting complex required for transport
EVs	extracellular vesicles
HuVECs	human umbilical vein endothelial cells
miR	microRNA
MSC (BM-) (AD-) (UC-)	mesenchymal stroma-/stem-like cells (bone marrow-) (adipose tissue-derived) (umbilical cord)
NTA	nano tracking analysis
TME	tumor microenvironment
